# Spatial Gradient of Axon Terminal Maturation Reveals the Sequential Active Zone Assembly in Adult *Drosophila* Mushroom Bodies

**DOI:** 10.17912/micropub.biology.001715

**Published:** 2025-08-15

**Authors:** Hongyang Wu, William Constance, Ayako Abe, Shu Kondo, Darren Williams, Hiromu Tanimoto

**Affiliations:** 1 Graduate School of Life Sciences, Tohoku University, Sendai, Miyagi, Japan; 2 Centre for Developmental Neurobiology, Institute of Psychiatry, Psychology & Neuroscience, King's College London, London, England, United Kingdom; 3 Department of Biological Science and Technology, Tokyo University of Science, Tokyo, Tokyo, Japan

## Abstract

Presynaptic active zones (AZs) form during synaptogenesis and are critical for ensuring precise synaptic transmission. Although much is known about the development of AZs at the neuromuscular junction, their assembly in the *Drosophila* central nervous system (CNS) remains incompletely understood. Here, we demonstrate the mushroom body α/β Kenyon cells in young adults in which new AZs are continuously formed, as a novel system to dissect the sequential AZ assembly in the CNS. With this system we show proof of principle using a tagged allele of the cell-adhesion molecule Neurexin-1 to investigate the structural maturation of synapses in the fly brain.

**Figure 1. Dissect the sequential active zone assembly in the adult Drosophila brain:**
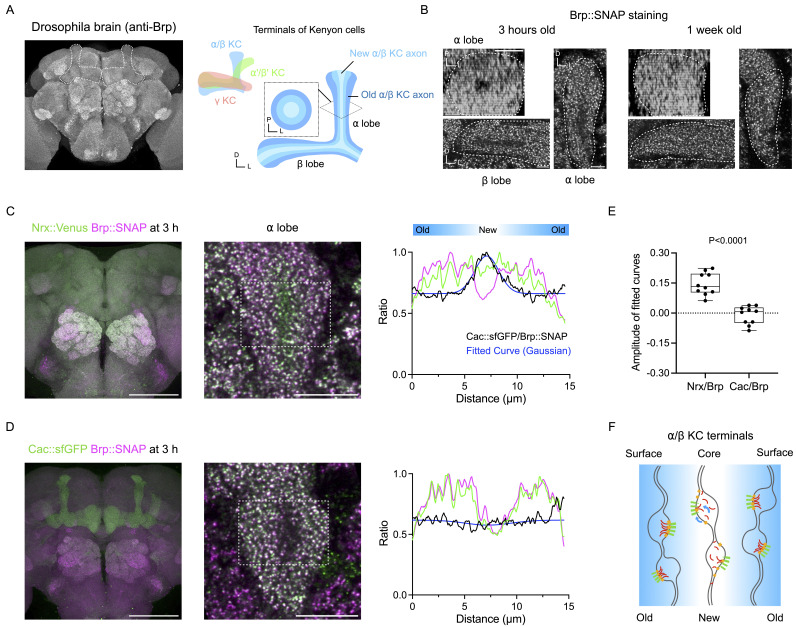
(A) α/β Kenyon cell axon terminals in the adult *Drosophila* mushroom bodies. Left: adult brain stained by anti-Brp antibody (nc82) immunostaining, dashed lines indicate the mushroom bodies. Right: schematic showing the organization of axon terminals of different Kenyon cell subtypes in the mushroom bodies. Newly α/β Kenyon cell axon terminals are formed in the core of the α/β lobe, displacing older ones towards the lobe surface. D, dorsal. L, lateral, P, posterior. (B) Brp signal is weak in the core of the α/β lobes in 3 h old adults, but not in 1 w old adults. Representative optical sections showing Brp::SNAP in the α/β lobes at 3 h and 1 week after eclosion. Dashed line indicates the lobes. Scale bar, 5 μm. (C) Representative images showing Nrx::Venus and Brp::SNAP in the brain of a 3 h old adult. Dashed line indicates the area where the intensity profiles are plotted. Green and magenta curves indicate the raw intensity of Nrx::Venus and Brp::SNAP respectively, normalized by the maximum value. Black curve indicates the ratio between Nrx::Venus/Brp::SNAP. Blue curve indicates the fitted Gaussian curve. Scale bars, brain, 100 μm; α lobe, 10 μm. (D) Representative images showing Cac::sfGFP and Brp::SNAP in the brain of a 3 h old adult. Dashed line indicates the area where the intensity profiles are plotted. Green and magenta curves indicate the raw intensity of Cac::sfGFP and Brp::SNAP respectively, normalized by the maximum value. Black curve indicates the ratio between Cac::sfGFP/Brp::SNAP. Blue curve indicates the fitted Gaussian curve. Scale bars, brain, 100 μm; α lobe, 10 μm. (E) Comparison of amplitude between Nrx::Venus/Brp::SNAP intensity ratio curves and Cac::sfGFP/Brp::SNAP intensity ratio curves. For both groups, n = 10. Mann-Whitney test, P<0.0001. (F) Schematic model of the AZ assembly in the α lobe. Green, red, and yellow components indicate Nrx, Brp and Cac respectively. In newly formed axon terminals in the core of the α/β lobe, Nrx accumulates to nascent AZs prior to Brp and Cac.

## Description

In presynaptic terminals, AZs are composed of evolutionarily conserved molecular machineries that regulate the synaptic vesicle exocytosis (Emperador-Melero & Kaeser, 2020; Südhof, 2012; Takamori et al., 2006). Studies of *Drosophila* larval neuromuscular junctions (NMJs) have revealed a sequential recruitment of AZ components (Fouquet et al., 2009; Owald et al., 2010, 2012; Vactor & Sigrist, 2017), and suggest that late-arriving materials are associated with AZ release probability (Akbergenova et al., 2018, 2025). However, the order of AZ component recruitment in the developing CNS remains largely uncharacterized due to technical challenges in performing long-term live imaging. Here, we demonstrate the adult mushroom body α/β Kenyon cells as a novel system to dissect the sequential AZ assembly in the CNS. α/β Kenyon cells are born after puparium formation and are generated until 8 days after larval hatching, about one day before the eclosion. Their axon terminals form the α/β lobes of the mushroom bodies where new axon terminals are found to be added to the core of the lobes, displacing older terminals toward the lobe surface (Lee et al., 1999; Lin, 2023). The manner in which the α/β lobes develop thus enables axon terminals at different developmental stages to be visualized within a single optical section of the lobes ( Fig. 1A ).

As such, we first asked whether AZs in these axon terminals develop at different times. To address this, we compared the levels of the active zone marker Bruchpilot (Brp) between newly eclosed adult flies (age < 3 hours (h)) and 1 week (w) old flies, using the SNAP-tagged allele (Kohl et al., 2014). We found that Brp::SNAP signals were weaker in the core than in the surface of the α/β lobes in adults 3 h after eclosion, while this difference is absent in 1 week old adults ( Fig. 1B ). This observation supports a model of an age difference in the presynaptic terminal assembly within the α/β lobes, where early-arriving components would fill the entire α/β lobe ahead of those that arrive later. We propose this system as a useful model for dissecting temporal features of AZ assembly in the CNS.

As a proof of principle, we used this system to analyze the synaptic recruitment of two key presynaptic components, Neurexin-1 (Nrx)—a cell-adhesion molecule implicated in synaptogenesis (Constance et al., 2018; Dean et al., 2003; Graf et al., 2004; Li et al., 2007; Owald et al., 2012), and Cacophony (Cac)—the α subunit of voltage-gated calcium channels (Cunningham & Littleton, 2023; Kawasaki et al., 2000). We first characterized the arrival of Nrx relative to Brp. We generated the Nrx::Venus knock-in fusion and visualized the expression of Nrx in the α/β lobes of 3 h old adults, together with Brp::SNAP. We found that Nrx::Venus signals were evenly distributed across the α lobe while Brp::SNAP signals were low in the core ( Fig. 1C ). The signal intensity ratio between Nrx::Venus and Brp::SNAP was therefore higher in the core than in the surface ( Fig. 1C ), suggesting that Nrx::Venus arrives to presynaptic terminals prior to Brp::SNAP.

Next, we analyzed the arrival of Cac relative to Brp in 3 h old adults. In contrast to Nrx::Venus, we found that endogenous Cac labeled with sfGFP (Cac::sfGFP) (Gratz et al., 2019) exhibited a signal pattern similar to Brp::SNAP (Fig 1D). The signal intensity ratio between Cac::sfGFP and Brp::SNAP was therefore consistent across the α lobe ( Fig. 1D ), suggesting similar arrival timing of Cac and Brp to developing AZs. Finally, to quantitatively compare the arrival timing between Nrx::Venus and Cac::sfGFP, we fitted their intensity ratio curves against Brp::SNAP using a four-parameter Gaussian model and measured the amplitude of the curves ( Fig. 1C- D). This analysis revealed significantly greater amplitudes for Nrx::Venus/Brp::SNAP intensity ratio curves than Cac::sfGFP/Brp::SNAP curves ( Fig. 1E ), indicating a larger arrival timing difference between Nrx and Brp. Collectively, these results suggest that Nrx accumulates at nascent AZs ahead of both Brp and Cac ( Fig. 1F ).

Nrx is long proposed to play a crucial role in recruiting AZ proteins when pre- and postsynaptic membranes make contact during synaptogenesis (Dean et al., 2003; Graf et al., 2004; Li et al., 2007). Consistent with this, we find that Nrx is present within the axon terminals prior to both Brp and Cac in the mushroom body α/β Kenyon cells, supporting its proposed role in the *Drosophila* CNS. Our results also suggest that Brp and Cac are recruited to nascent CNS AZs at approximately the same time, in agreement with previous observations (Fouquet et al., 2009). With the recent development of fluorescence labeling and epitope tagging tool kits (Kondo et al., 2020; Nagarkar-Jaiswal et al., 2015; Venken et al., 2011), future studies can go beyond examining the recruitment of just AZ components. The maturation of nanoscopic synaptic structures such as the postsynaptic receptor segregation (Akbergenova et al., 2018) and the development of AZ molecular organization are of great interest (Ghelani et al., 2023; Mrestani et al., 2021). Given the advantage of working with fixed samples, our simple but powerful system offers the possibility of interrogating these presynaptic structures using super-resolution and expansion microscopy.

## Methods

**Fly husbandry**

Flies were maintained on standard cornmeal food at 25 °C under a 12:12 h light-dark cycle for all experiments. For newly hatched (3 h old adult) group, flies were collected within 3 h after eclosion and dissected immediately after collecting. For 1 week old group, flies were flipped every three days and were dissected 1 week after eclosion.

**Transgenic flies**

CRISPR/Cas9-mediated homologous recombination was used to insert Venus downstream of the transmembrane domain of the endogenous Nrx-1 protein. The donor vector contained a left homology arm of 500 bp, Venus, a floxed 3xP3-RFP marker and a right homology arm of 500 bp. A guide RNA sequence (ATCCATTGGTGCTAGTATTT) targeting the Nrx-1 locus was cloned into pBFv-U6.2 (Kondo & Ueda, 2013) to generate a gRNA expression vector. The donor and gRNA vectors were co-injected into fertilized eggs carrying the *nos-Cas9* transgene (Kondo & Ueda, 2013). Successful transformants were identified by eye-specific RFP fluorescence. Flies were then crossed to a TM6B-Cre balancer to remove the RFP marker before use.

**Sample preparation**

Flies were anesthetized on ice before dissection. The dissection was performed in cold PBS solution to collect brain samples. Brain samples are fixed in 2% paraformaldehyde for 1 hour then washed 3×20 min in 0.1% PBT (0.1% Triton X-100 in PBS). For immunohistochemistry (anti-Brp staining), samples are incubated in 3% normal goat serum (NGS; Sigma-Aldrich; G9023) for 1 hour at room temperature. Samples were incubated in antibody solutions at 4°C for 48 hours for both primary (anti-Brp, DSHB, Cat#nc82; RRID: AB_2314866) and secondary antibodies (AlexaFluor-568 goat anti-mouse, Invitrogen, Cat#A11004; RRID: AB_2534072). For SNAP chemical tagging, samples were incubated in SNAP-Surface 546 (1:10000; NEB; S9132S) in 0.3% PBT for 15 min and washed 3×20 min in 0.1% PBT. Samples are mounted on microscope slides using the mounting medium SeeDB2G or SeeDB2S according to the imaging condition (Ke et al., 2016).

**Confocal imaging**

Images were acquired using the Olympus FV1200 confocal microscope platform equipped with GaAsP high-sensitivity detectors. For anti-Brp immunostaining in Fig. 1A, the 30×/1.05 NA objective (UPLSAPO30XS, Olympus) was used; 559 nm laser power: 0.3%; voxel size: 0.414 × 0.414 × 0.82 µm. For Brp::SNAP staining and images in Fig. 1B- D, the 60×/1.42 NA objective (PLAPON60XO, Olympus) was used; 472 nm laser power: 1.5%, 559 nm laser power: 1.0%; voxel size: 0.79 × 0.79 × 0.37 µm. For images used in the quantification in Fig. 1E, the 40×/1.30 NA objective (UPLFLN 40X, Olympus) was used; 472 nm laser power: 1.5%, 559 nm laser power: 1.0%; voxel size: 0.31 × 0.31 × 0.54 µm.

**Data analysis**

Intensity profiles were generated by using the plot function in Fiji. For every sample, a square area was selected to frame the α lobe in a z-plane where both the core and the surface of the α/β lobe are displayed. The center of the area was aligned to the center of the core. The intensity profile of the frame was plotted, with the x-axis indicating the distance and the y-axis indicating the average grey value of pixels. Data of the intensity profile was then fitted with a four-parameter gaussian model: Y = Y0 + A*exp(-((X - Xc)^2) / (2 * SD^2). Y0 is the baseline offset (minimum value), A is the peak amplitude (maximum value), Xc is the center (peak position), SD is the standard deviation. Curve-fitting was performed in Prism GraphPad 10, where Y0 was constrained to be constantly equal to the average value within a certain range (for Fig. 1C- D, X = 0 to X = 2.054 mm; for Fig. 1E, X = 0 to X = 2.17 mm). Xc was constrained to be constantly equal to the average of all X values when fitting Cac::sfGFP/Brp::SNAP profiles, but not for Nrx::Venus/Brp::SNAP. The absolute value of A was constrained to be less than 1. SD has no constraint.

## Reagents

**Table:** 

Fly line	Identifier/Reference
*brp::SNAP*	(BDSC_58397) (Kohl et al., 2014)
*cac::sfGFP*	(Gratz et al., 2019)
*nrx::Venus*	This study
